# A Rebuttal of the 12 Breast Reconstruction Points to Minimize Implant Contamination

**DOI:** 10.1097/GOX.0000000000002210

**Published:** 2019-05-17

**Authors:** Eric Swanson

**Affiliations:** From the Swanson Center, Leawood, Kans.

## Sir,

Cagli et al^1^ adapt the 14-point plan^2,3^ to 165 women undergoing breast reconstruction with implants but report no data. The authors reportedly use 12 of the 14 points in most cases, but there are many exceptions. They describe an S-shaped incision, dissecting close to the nipple/areola complex (point 2).^1^ Nipple shields (point 3) are irrelevant to women undergoing a full mastectomy. The authors acknowledge that mastectomy is a traumatic dissection, so that a “careful atraumatic dissection” (point 4) is impractical. The breast parenchyma is dissected during any partial mastectomy (point 6). A dual plane (point 7) is not applicable to patients whose lower pole breast tissue has been removed; a subglandular plane no longer exists. An introductory sleeve (point 9) would not be advantageous in a breast reconstruction with wide exposure. The surgeon must still touch the implant to position it properly. Breast tissue is not sterile, but rather inhabited by commensal bacteria.^4^ The authors use drains (point 12) to avoid seromas. With these exceptions, the point total is reduced to 7.

Are the 7 remaining points valid? Almost all (99%^5^) plastic surgeons already routinely administer preoperative intravenous antibiotics (point 1). Performing “careful hemostasis” (point 5) is simply good surgical practice and not particularly relevant to bacterial contamination. Recent systematic reviews fail to show a clear benefit for triple antibiotic or betadine irrigation (point 8).^6–8^ In fact, triple antibiotic pocket irrigation may be counterproductive.^8,9^ Betadine may be nonsterile^10^ and should not be used to irrigate surgical wounds.^10,11^ Washing a sterile breast implant in its box cannot make it “more sterile.”^12^

Minimizing implant exposure to the air (point 10) does not make sense. Sterile instruments are exposed to the air in any sterile operation and are not changed routinely absent a break in sterility. Regardless, it is normal practice to open an implant container when it is time to insert the implant. Changing gloves and instruments (point 11) implies that the existing gloves and instruments are already contaminated, so the damage has already been done, so to speak. Logically, there is no reason to exchange sterile gloves and instruments for new sterile gloves and instruments.

Using a layered wound closure (point 13) reduces the risk of dehiscence. Like careful hemostasis, a layered wound closure is simply part of normal surgical practice. Future antibiotic prophylaxis for procedures that breach the skin or mucosa (point 14) is not recommended consistently by surgeons using the 14-point plan (fortunately).^3^ Unnecessary antibiotic use can lead to an opportunistic infection or allergic reaction and is known to promote antibiotic resistance in the community.^13^

Only one factor (not one of the points) is known to be linked to breast implant-associated anaplastic large-cell lymphoma—textured implants.^14^ Smooth implants offer a safe alternative. It is clear that the problem is a faulty product,^14,15^ not faulty surgeon technique, a notion that is driven by conflicted investigators.^14^ Implant manufacturers often fund microbiological studies that identify an infectious etiology,^2^ or simply pay the article processing fee.^1^ Evidence-based medicine must take precedence over unscientific formulas.
